# Climatic and regional antibiotic resistance patterns of *Staphylococcus aureus* in South African dairy herds

**DOI:** 10.4102/ojvr.v86i1.1674

**Published:** 2019-07-10

**Authors:** Joanne Karzis, Inge-Marie Petzer, Edward F. Donkin, Vinny Naidoo, Eric M.C. Etter

**Affiliations:** 1 Department of Production Animal Studies, Faculty of Veterinary Science, University of Pretoria, Pretoria, South Africa; 2 Department of Animal and Wildlife Sciences, University of Pretoria, Pretoria, South Africa; 3 Department of Research & Postgraduate Studies; University of Pretoria, Pretoria, South Africa; 4 Biomedical Research Centre, Faculty of Veterinary Science, University of Pretoria, Pretoria, South Africa; 5 CIRAD, UMR Animal, Santé, Territoires, Risque et Ecosystèmes (ASTRE), Montpellier, France; 6 ASTRE, University Montpellier, CIRAD, INRA, Montpellier, France

**Keywords:** antibiotic resistance, *S. aureus*, mastitis, seasons, regions, dairy cattle

## Abstract

South Africa is a large country of approximately 1.22 million km^2^, made up of nine provinces with three climatic zones. Farming in the country is mostly defined by regional differences. Of the different organisms isolated from milk samples of dairy cows, *Staphylococcus aureus* poses a challenge to maintain udder health and wholesome dairy products for human consumption. Antibiotic resistant bacteria are therefore a potential health hazard. The objective of this study was to investigate the seasonal and regional relationships of antibiotic resistance of *S. aureus*, of which little is known. This study was undertaken to evaluate a data set of 3410 *S. aureus* isolates, taken from milk samples with a somatic cell count of > 400 000 cells/mL from commercial dairy herds. These isolates were tested for antimicrobial susceptibility using the Kirby Bauer method for ampicillin, cloxacillin, penicillin G, clindamycin, oxy-tetracycline, cephalexin, cefuroxime and tylosin. The samples were from 830 dairy herds, out of the estimated 2000 commercial dairy herds in South Africa. All the antibiotics tested, except for cephalosporins, showed a predicted prevalence of resistance of above 50% in most provinces, which is a concern. The lowest prevalence of resistance to the majority of the categories of antibiotics tested was present in KwaZulu-Natal during spring. The cephalosporins had the lowest levels of prevalence of bacterial resistance in Gauteng during winter. Resistance patterns of *S. aureus* to the eight antibiotics varied in the different seasons and provinces, possibly because of different weather conditions, and the action and spectrum of antibiotics.

## Introduction

Mastitis remains the most important economic disease in dairy cattle in first world countries despite the progress made in improving general udder health in recent years. The discovery and use of antimicrobial agents in the 20th century has been one of medical science’s greatest achievements. However, bacteria are becoming increasingly resistant to these agents (Roberts 2002). Bacterial antimicrobial resistance in humans is interlinked with antimicrobial resistance in other populations, especially farm animals, which are exposed to enormous quantities of antibiotics (despite attempts at reduction) and which act as another reservoir of resistance genes (Woolhouse et al. 2015).

**FIGURE 1:**
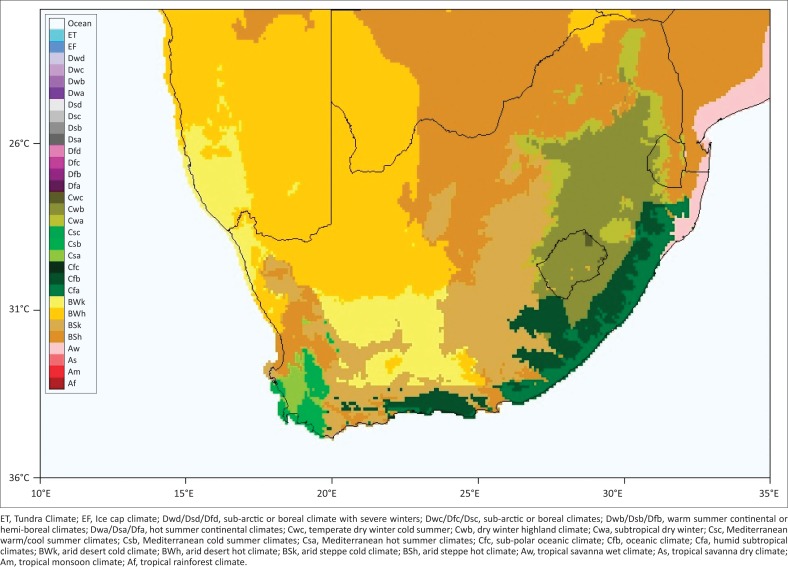
Map of South Africa with the Köppen-Geiger climate classification.

According to Wingfield and Kenagy (1991) and Blank (1992), seasonal changes are cyclic, largely predictable and represent the strongest and most abundant source of external variation influencing human and natural systems. Although generalisations can be made about the climate in the various provinces, there are considerable variations within each province. KwaZulu-Natal has a subtropical climate with mostly summer rainfall, while the Western Cape has a Mediterranean climate with mostly winter rainfall. The Eastern Cape has generally dry and cold winters and hot summers, but with rain mostly in late summer (Smith 2006). The Free State has mostly summer rainfall, and late summer rainfall in some parts, with cold dry winters. The Limpopo province has a hot dry winter climate with summer rainfall. Mpumalanga is a summer rainfall area, divided into the Highveld with cold winters and the Lowveld which has a subtropical climate similar to that of KwaZulu-Natal. Gauteng and the North West province have cold dry winters and hot summers with rainfall (Smith, 2006). The incidence and duration of frost occurs in winter in the higher altitudes of the Western Cape, Eastern Cape, Free State, Gauteng, KwaZulu-Natal, Mpumalanga and Limpopo, rather than in the rest of the country (Smith 2006). The Western Cape, Eastern Cape, Free State and North West provinces all experience lower average temperatures than those in Gauteng and the Mpumalanga Highveld, but the Limpopo province has the highest average temperatures (Smith 2006). A commonly used climate classification map is that of Wladimir Köppen (Kottek et al. 2006) which was used to develop a climate classification map for South Africa (Figure 1). This shows the extreme variability of the weather and climate across different parts of South Africa.

In South Africa, intramammary remedies are available by prescription from a veterinarian according to *Act 101* of 1965, but also without prescription under *Act 36* (*Stock Remedies Act*); and this enables producers to buy such products freely, which may lead to incorrect use of these products and an increase in antibiotic resistance. This unique situation explains the necessity of such a study as this, concerning antibiotic resistance, and perhaps a review of the relevant legislation.

In South Africa, little information is available on the variation of the prevalence of antibiotic resistance of *Staphylococcus aureus* (in different seasons and regions). Such knowledge will have an important impact on the treatment of animals with antibiotics, especially in South Africa with the unique climatic variations of the environment amongst the different provinces of the country.

The aims of this study were to identify the seasonal weather effects in nine provinces, in relation to the prevalence of *S. aureus* resistance to eight antibiotics that had been tested in South African dairy herds over an 11-year study period.

The objectives included the calculation of the prediction of the prevalence of resistance for the different antibiotics in different seasons and provinces; a comparison of the proportions of the prevalence of bacterial resistance between all antibiotics used; the creation of a time series to show trends of resistance over time; as well as to identify any relationship between seasons and or regions and the prevalence of bacterial antibiotic resistance.

## Methods and materials

**FIGURE 2:**
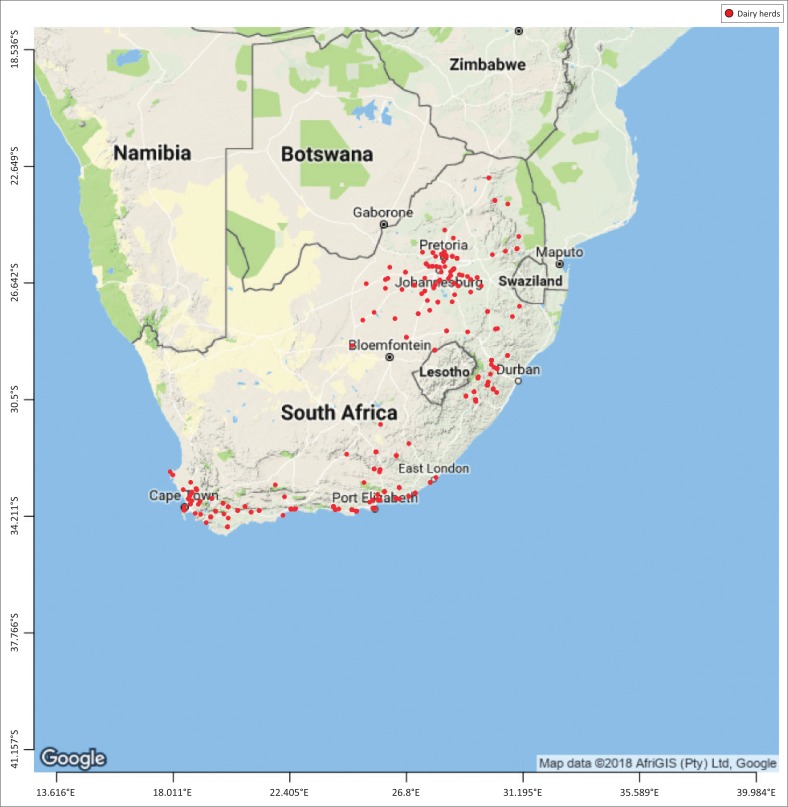
Distribution of milk sampling locations in South Africa.

From 2000 to 2010, a total of 3410 susceptibility tests were performed on *S. aureus* isolated from milk samples of commercial dairy herds in South Africa. *Staphylococcus aureus* was chosen for this study as it is one of the most important mastitis-causing bacteria. The samples represented 830 dairy herds, out of the total of approximately 2000 commercial dairy herds in the nine provinces of South Africa, namely Gauteng (*n* = 301), KwaZulu-Natal (*n* = 369), Free State (*n* = 67), Eastern Cape (*n* = 464), Western Cape (*n* = 646), Northern Cape (NC) (*n* = 4), North West (*n* = 52), Limpopo (North) (*n* = 56) and Mpumalanga (*n* = 68) (Figure 2).

The majority of these dairy producers send milk samples to the Milk Laboratory, Faculty of Veterinary Science, University of Pretoria (FVS-UP), for testing (microbiology and cytology) on a routine basis, as part of a proactive udder health management programme. The minority of producers that do not test on a routine basis may be those that test when a crisis occurs in the udder health of their herds. The milk samples were collected either by the farmers themselves according to a standard operating procedure (NMC 2004) or by professional milk samplers.

All samples were analysed at the Milk Laboratory (FVS-UP). Microbiological and cytological examinations were performed on all milk samples (NMC 2004). Isolates tested for antimicrobial susceptibility were all selected from milk samples with a somatic cell count (SCC) (Fossomatic 5000, Rhine Ruhr) of more than 400 000 cells/mL (NMC 2004), in order to include cases of subclinical mastitis. The Kirby Bauer method (Bauer et al. 1966) with published breakpoints was used to determine antimicrobial susceptibility. The results were based on the diameter of the inhibition zones and were classified as sensitive, intermediate or resistant in accordance with the Clinical and Laboratory Standard Institute (CLSI 2008, CLSI 2012).

Eight antibiotics used in intramammary treatment (dry and lactating remedies) that are available in South Africa were tested. These were beta-lactams (ampicillin 10 *µ*g, cloxacillin 5 *µ*g, penicillin G 10 IU), cephalosporins (cephalexin 30 *µ*g, cefuroxime 30 *µ*g), lincosamides (clindamycin 10 *µ*g), tetracyclines (oxy-tetracycline 30 *µ*g) and macrolides (tylosin 30 *µ*g). The South African national average daily milk yield was 20.2 kg in 2013, and average yield varied slightly over the years of the study period; and it would have varied between herds, within herds between cows, and in different stages of lactation (Lactodata 2013). The lactating cow numbers of the herds used varied from approximately 30 (smallest herd) to 1700 cows (largest herd), with an average of about 100–400 lactating cows tested during the period of this investigation.

The producers in this study were part of the proactive udder health programme (Karzis et al. 2018). Antibiograms were performed with every routine herd examination and the results were used by the veterinarians and producers when deciding on antibiotic treatment which was administered by trained staff. As part of the proactive udder health programme, *S. aureus* positive cows were identified, placed in a separate group for life, milked last and culled in due course (Petzer et al. 2009).

### Statistical analysis

Data from the NC province were removed from analysis as they were not sufficient compared to the information available from other provinces. The results were grouped into two categories as: resistant or susceptible. Results that were originally listed as being intermediate (CLSI guidelines) were grouped together with the resistant results in this analysis, as this could only accommodate for two categories.

Comparisons of proportions of resistance of antibiotics using the Bonferroni *p*-value adjustment method (Benjamini & Hochberg 1995) were done to compare differences in proportions of resistance of *S. aureus* to the different antibiotics between all the antibiotics used in all provinces and for all seasons over time.

Time series analysis was used to identify both seasonality and the trend of antibiotic resistance of *S. aureus* over time for the eight antibiotics used. The ‘zoo’, ‘xts’ and ‘forecast’ packages of the R software^©^ (version 3.3.3 for Mac) were used to perform these time series analyses. Data were averaged per month. Missing data were filled using the seasonal Kalman filter. A local polynomial regression smoothing (LOESS ‘LOcal regrESSion’) was applied to the data in order to extract first the effect of seasonality. The seasonal component was removed from the data and the same method was applied to obtain the trend (Cleveland et al. 1990). The final result of the stepwise analysis of the data into seasonal, trend and remainder components was obtained after several iterations of this process. To test the goodness of fit of the stepwise analysis, the autocorrelation function of the ‘remainders’ (residues) was analysed to check if they were stationary. An augmented Dickey–Fuller test (ADF test) was used to confirm stationary residues compared to the null hypothesis which was that the time series had a unit root.

Further apparent relationships between season and province on the prevalence of antibiotic resistance of all *S. aureus* isolates were tested with a general linear mixed model (GLMM) (‘glmer’ within ‘lme4’ package with R software^©^ version 3.3.3) using a logit link-function. This model allowed the random effect from the different herds and from the repetition of data collection over time, to be taken into account. This type of GLMM takes into account this random effect when comparing the different provinces and the different seasons as well as the interactions between seasons and provinces. The existence of interaction between provinces and seasons was checked by using logistic regression. In case of the existence of these interactions, all the potential interactions were compared with the specific interaction showing the lowest level of antibiotic resistance prevalence (according to univariate pre-analysis). Odds ratios (OR), their confidence interval and the associated *p*-value (using the threshold *p* = 0.05 for statistical significance) were calculated from the results of the GLMM. When no statistically significant interaction was found, the GLMM was re-run to remove the interaction and to compare seasons and provinces, respectively, using for both of them the specific seasons and or provinces showing the lowest antibiotic resistance prevalence as reference (according to univariate pre-analysis). Comparisons were also based on OR calculated from the GLMM using the following formula:

$$\begin{array}{l} \text{OR}=\exp(\text{Estimate}),\\ {\text{OR-IL}}_{95}\%=\exp(\text{Estimate-}1,96*\text{se}),\\ \text{OR-}\exp(\text{Estimate}+1,96*\text{se}), \end{array}$$ [Eqn 1]

where ‘Estimate’ is the estimate of the fixed effect obtained from the GLMM and ‘se’ the standard error associated with the estimate of the fixed effects. LL_95_% and UL_95_% are the lower limit of the 95% confidence interval and the upper limit of the 95% confidence interval, respectively.

To calculate the prevalence of resistance for each season, province or interactions and their confidence intervals with an accepted error of 5% the results of the GLMM could not be used directly. Indeed, the formula applied on the results of the ‘glmer’ function to obtain the respective prevalence of resistance for all seasons, provinces or for all the potential interactions does not take into consideration the random effect. Therefore, the prediction of level of resistance was used, using the GLMM as a model applied to the complete data set based on the assumption that the data set was truly a good representative sample of the population of interest. The ‘predict’ function of the ‘lme4’ package adapted for fitted mixed-effect models was used to calculate the prevalence of resistance per province, per season or per province within season.

The data analysis accounted for all seasons: spring (*n* = 482) (01 September to 30 November); summer (*n* = 404) (01 December to 28 February); autumn (*n* = 530) (01 March to 31 May); winter (*n* = 607) (01 June to 31 August). This is a broad classification as used as by the weather services, although definitions of seasons and rainfall patterns can be variable.

### Ethical considerations

This article followed all ethical standards for a research project without direct contact with human or animal subjects.

## Results

In this study, the overall comparison of proportions of resistance of *S. aureus* between the antibiotics investigated was significantly different for all pairs indicated, except for penicillin G and ampicillin, clindamycin and ampicillin and for oxy-tetracycline and cloxacillin. Of the eight antibiotics tested, tylosin and penicillin G showed the highest prevalence of resistance (67.1% and 50.3%, respectively), cefuroxime and cephalexin the lowest (14.39% and 23.05%, respectively) (Table 1).

**TABLE 1:** *P*-values obtained from the pairwise comparison of proportions of prevalence between the eight antibiotics tested for antibiotic resistance of *Staphylococcus aureus* (over the 11 year period and in all seasons), using the Bonferroni adjustment method.

Antibiotics	Ampicillin	Cefuroxime	Cloxacillin	Penicillin G	Cephalexin	Clindamycin	Oxy-tetracycline	Tylosin
Cefuroxime	**	-	-	-	-	-	-	-
Cloxacillin	**	**	-	-	-	-	-	-
Penicillin G	1	**	**	-	-	-	-	-
Cephalexin	**	**	**	**	-	-	-	-
Clindamycin	0.23	**	**	**	**	-	-	-
Oxy-tetracycline	**	**	1	**	**	**	-	-
Tylosin	**	**	**	**	**	**	**	-
Resistance (%)	47.28	14.39	30.04	50.30	23.05	42.94	31.99	67.10

Note: Resistance, overall antibiotic resistance.

** , means *p*-value = <10^−5^.

From the time series analysis, the stepwise analysis of the data for all the antibiotics resulted in stationary remainders confirmed by the analysis of the correlogram and the ADF test. The trends, seasonal relationships of the data (repeated pattern per year) and remainders were calculated in the time series carried out for all eight antibiotics used.

**FIGURE 3:**
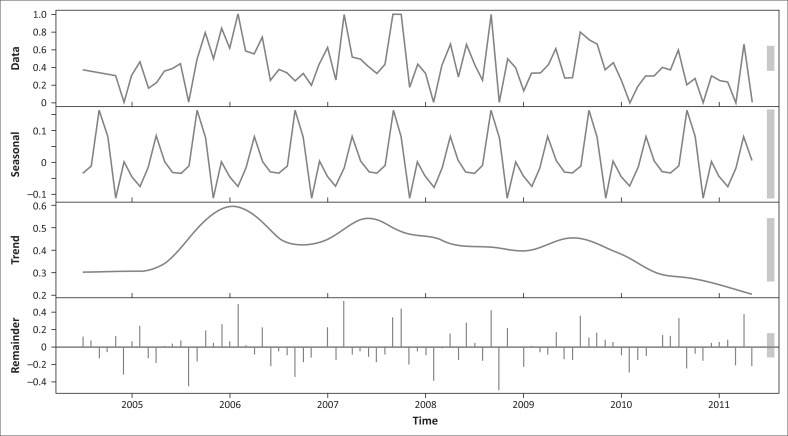
Time series of resistance of *Staphylococcus aureus* to ampicillin, showing the data, trend, seasonal effects and the remainder.

However, a meaningful trend from the time-series stepwise analysis was shown only for ampicillin (Figure 3).

Concerning the GLMM, we found that the repetition of the data collected over time did not have an effect, whereas there was a clear effect for all the antibiotics tested.

The GLMM applied to the data for *S. aureus* resistance to ampicillin, penicillin G, clindamycin and tylosin showed that there was no interaction between province (Table 2) and season (Table 3); thus, these were tested separately. In addition, no significant differences were shown between the prevalence of antibiotic resistance according to the season for tylosin.

**TABLE 2:** Prediction of expectation of *Staphylococcus aureus* antibiotic resistance to ampicillin, penicillin G, clindamycin and tylosin for the provinces showing significant differences according to the general linear mixed model.

Product	Province	Resistance (%)
Ampicillin	KZN†	34
	WC‡	56
Penicillin G	KZN†	37
	WC‡	57
Clindamycin	KZN†	23
	NW‡	58
Tylosin	KZN†	56
	L‡	83

KZN, KwaZulu-Natal; L, Limpopo; NW, North West; WC, Western Cape.

† , Indicates the province with the lowest prevalence of antibiotic resistance within which a significant difference existed.

‡ , Indicates the provinces which has significantly higher antibiotic resistance than KZN.

**TABLE 3:** Prediction of expectation of *Staphylococcus aureus* antibiotic resistance to ampicillin, penicillin G and clindamycin for different seasons where significant differences were shown according to the analysis using the general linear mixed model.

Product	Season	Resistance (%)
Ampicillin	Spring†	39
	Summer‡	48
Penicillin G	Spring†	41
	Summer‡	53
Clindamycin	Spring†	36
	Autumn‡	43

† , Indicates the season which showed the lowest level of antibiotic resistance within which a significant difference existed.

‡ , Indicates the seasons with significantly higher levels of antibiotic resistance than spring.

For the prevalence of antibiotic resistance for *S. aureus* to cefuroxime, cephalexin, oxy-tetracycline and cloxacillin, the GLMM analysis showed significant interactions between season and province (Table 4). The prevalence of *S. aureus* resistance to cloxacillin in winter was significantly higher than in spring, and the prevalence of antibiotic resistance in the North West and Free State provinces was also significantly higher than the prevalence of antibiotic resistance in KwaZulu-Natal.

**TABLE 4:** Prediction of expectation of *Staphylococcus aureus* of antibiotic resistance to cefuroxime, cephalexin, oxy-tetracycline and cloxacillin for different seasons and provinces where significant differences were shown according to the analysis using the general linear mixed model.

Product	Season	Province	Resistance (%)
Cefuroxime	Winter†	GP†	3
	Autumn‡	NW	33
Cephalexin	Winter†	GP†	8
	Summer‡	FS	73
Oxy-tetracycline	Autumn†	KZN†	12
		GP	61
		L	61
Cloxacillin	Spring†	KZN†	17
	Winter‡	FS	57
		NW	57

FS, Free State; GP, Gauteng; KZN, KwaZulu-Natal; L, Limpopo; NW, North West.

† , Indicates the season and province together which showed the lowest level of antibiotic resistance within which a significant difference existed.

‡ Indicates the seasons and provinces with significantly higher levels of antibiotic resistance.

The results presented in Tables 2–4 show the predictions according to the model with presentation of the significant associations of seasons or provinces or the interactions of seasons and provinces for each antibiotic investigated.

In KwaZulu-Natal during spring, the *S. aureus* isolates had the lowest prevalence of antibiotic resistance for clindamycin (lincosamide) and for the beta-lactam group (ampicillin, cloxacillin and penicillin G) when compared to all other provinces and seasons. Findings generated in KwaZulu-Natal during spring (lowest level of antibiotic resistance) were therefore used as a baseline against which the other provinces and seasons were compared (Tables 2 and 3). Conversely, cefuroxime and cephalexin showed a different trend to the rest of the antibiotics, by having the lowest prevalence of *S. aureus* antibiotic resistance in Gauteng and in winter (Table 4). Autumn in KwaZulu-Natal was the season that showed the lowest prevalence of *S. aureus* antibiotic resistance for oxy-tetracycline (Table 4). In addition, the prevalence of resistance of *S. aureus* had a direct relationship to season and provinces as well as the interaction of season and provinces.

## Discussion

The antibiotics used in this study differed in their action and also in their spectra. The beta-lactams are bactericidal, while clindamycin, oxy-tetracycline and tylosin are bacteriostatic. Ampicillin, cloxacillin, cefuroxime and oxy-tetracycline are broad spectrum antibiotics, while penicillin G, cephalexin, clindamycin and tylosin are active against gram-positive micro-organisms. There have been variations in the prevalence of *S. aureus* antibiotic resistance overall (Table 1) and in different provinces and different seasons (Tables 2–4). Reasons for these variations are unclear. Antibiotic resistance could also occur from random genetic mutations and subsequent natural selection of bacteria in order for them to survive.

The decreasing trend of antibiotic resistance found in a previous study conducted in South Africa (Karzis et al. 2018) was confirmed for some ampicillin, using the time series analysis in the current study (Figure 3). The lowest level of antibiotic resistance of *S. aureus* to beta-lactams and clindamycin was in KwaZulu-Natal during spring, which implied a significant positive effect or association of this season on the prevalence of resistance. This could possibly be because of the increased daily milk production potential of cows grazing on pastures as well as the calving patterns in KwaZulu-Natal in spring. A previous study, in which seasonal effects were not investigated, showed that the overall prevalence of mastitis did not change during the same 11-year study period (Karzis et al. 2018).

A possible reason for a lower prevalence of antibiotic resistance of *S. aureus* to cephalosporins during winter in Gauteng may be that the dry cold season has generally a lower probable prevalence of intramammary infections that would require less treatment. This would be supported by the occurring higher average incidence of frost duration in Gauteng (Smith 2006), which would have suppressed insect vectors associated with mastitis pathogen transmission (Zadocks et al. 2011). Mastitis prevalence in different seasons and provinces of South Africa must be investigated further in future to evaluate any possible association with bacterial antibiotic resistance. Mastitis has been known to increase in wet conditions because of muddy paddocks and bedding, which increases hygiene and management challenges during these periods. In this study, the highest number of samples used was from the main dairy producing provinces, namely KwaZulu-Natal, the Eastern Cape and Western Cape (Figure 2). Most provinces showed a prediction of the occurrence of bacterial resistance for *S. aureus* to these antibiotics of more than 50%, which is a concern (Tables 2–4).The lowest prevalence of resistance of *S. aureus* to oxy-tetracycline (12%) was shown in KwaZulu-Natal in autumn; and the lowest levels of resistance to tylosin (56%) were shown in KwaZulu-Natal, with no apparent difference between seasons. Tylosin is one of the few intramuscular products registered for use in the treatment of mastitis in South Africa, which has been found not to be effective.

The highest prevalence of *S. aureus* resistance against penicillin G and ampicillin was indicated during the hot summer months in South Africa (Table 3). The only antibiotic that showed an increase in the prevalence of resistance in *S. aureus*in in winter was cloxacillin in the North West and the Free State (Table 4). However, these observed effects were only on a small group of antibiotics (ampicillin and penicillin G), compared to the overall number of eight antibiotics used in this study. Thus, these observed effects probably indicated no general effect. It is difficult to account for the reasons causing these differences.

The data also showed that most of the identified resistance of *S. aureus* was to the beta-lactam group. These are the products mostly used currently as intramammary remedies in South Africa (as there is only a small selection of remedies available) (Eagar, Swan & Van Vuuren 2012).

The seasonal and regional effects on antibiotic resistance in South Africa cannot be compared directly to studies in other countries, as climatic conditions, rainfall patterns and related management systems differ. Based on the findings of this study on seasonal and regional differences, veterinarians in practice should rather use local surveillance data in preference to national or international data.

In addition to the different weather and ecological conditions (Figure 2), there are also differences in feeding systems [such as with total mixed rations (TMRs)] where cows are kept both in free-stall barns and camps or on pastures: the number of milkings per day and in the average herd size in the different provinces (468 in the Eastern Cape, 367 in KwaZulu-Natal and 203 in the Western Cape, with herd size in the rest of the country ranging between 96 and 175) (Lactodata 2013).

KwaZulu-Natal, the Eastern Cape and Western Cape have mostly pasture-based herds that are milked twice per day. In the rest of South Africa, the high producing large herds are mostly herds fed TMRs which are milked three times per day. This may have had an effect on antibiotic efficiency and the development of resistance because the intramammary products widely available in South Africa are mostly time-dependant. Three times a day milking could also have had an effect on the prevalence of mastitis pathogens, as the cows are handled more frequently (eight hourly), and have the potential to be exposed to more pathogens in the parlour if the milking hygiene is not excellent. This would explain the results of this study, which showed that in KwaZulu-Natal (where herds were generally milked twice per day and mainly pasture fed), there was the lowest level of resistance for almost all antibiotics tested. The different usage of intramammary antibiotics (lactating and dry cow therapies) in the different provinces could also possibly have contributed to a difference in bacterial antibiotic resistance in different provinces. Unfortunately, these data were not made available by suppliers. A study by Fox et al. (1995) also found that location, herd and season significantly influenced prevalence of intramammary infections, which is also implied in this study which found that prevalence of antibiotic resistance can be influenced by provinces and seasons.

Challenging weather conditions and climatic differences may cause bacteria to form protective mechanisms such as biofilm which might cause an increase in the prevalence of antibiotic resistance (Da Silva Meira et al. 2012; Melo et al. 2014). Many normal microbiota bacteria produce a capsular polysaccharide matrix (glycocalyx) to form a biofilm. A biofilm is a system that can be adapted to changing environmental conditions and constitutes a physical barrier, which protects the encased bacteria from detergents and sanitizers (Potera 1999). The hypothesis is that the variation in prevalence of antibiotic resistance of bacteria between seasons that show extreme weather conditions may be because of the variation in the population of bacteria that can produce biofilm in the different seasons. The findings of this study might be related to such a hypothesis.

The ability of *S. aureus* strains to produce biofilm and conditions enhancing this production have not been studied under South African conditions in the different seasons and provinces.

According to Atulya et al. (2014), an acidic pH has a positive correlation with biofilm formation. The current intramammary antibiotics available for use for mastitis in South Africa include highly acidic antibiotics such as penicillins, which could favour the formation of biofilm and the establishment of infections in udder tissue. However, lipid soluble (non-ionised) antibiotics such as clindamycin are able to penetrate the biofilm (Kundukad et al. 2017). The ionised form of an antibiotic has low lipid solubility (but high water solubility) and high electrical resistance, and thus cannot penetrate cell membranes easily (e.g. ampicillin, cloxacillin, penicillin G and oxy-tetracycline) (Shargel et al. 2012). Evidence indicates that photoperiod-driven physiologic changes are typical in mammalian species, including some in humans (Dowell 2001). These are examples of how the environment affects survival of organisms in general.

Future studies need to be conducted to determine antibiotic resistance genes present in *S. aureus* isolated from dairy cattle in South Africa, as new *S. aureus* strains have been identified (Monistero et al. 2018). In addition, the determination of biofilm expression of these isolates should be checked for seasonal and regional patterns. Other factors such as calving patterns and parlour design that may also contribute to the development of mastitis and antibiotic resistance in different seasons and regions also need to be investigated.

## Conclusion

There were seasonal and regional apparent effects on the prevalence of antibiotic resistance in *S. aureus* in South Africa.

Reasons for these associations may be attributed to factors such as the different weather, ecological and rainfall patterns and differences in management systems, such as milking intervals during the different seasons and in different provinces, as well as the feeding methods used in different provinces. Resistance patterns were also found to differ between bacteriostatic and bactericidal antibiotics depending on spectrum (narrow vs. broad).

Management and hygiene challenges are increased under the warmer and wetter more challenging conditions in the various South African provinces and at different times of the year.

It is of concern that all the antibiotics tested, except for cefuroxime, showed a predicted prevalence of resistance of above 50% in most provinces. The lowest predicted prevalence of resistance was in KwaZulu-Natal in spring for all antibiotics tested except for the cephalosporins which was in Gauteng during winter.
